# CardioGAP+: bridging the gap between patient identification and therapy implementation in cardiovascular risk

**DOI:** 10.3389/fcvm.2026.1881174

**Published:** 2026-07-22

**Authors:** João Fonseca Sousa, Bárbara Rocha, Marta Silva, Mariana Canelas-Pais, Alice Coelho, Irene Guimarães, Vanessa Queirós, Rui Baptista

**Affiliations:** 1 Cardiology Department, Unidade Local de Saúde de Gaia e Espinho E.P.E., Vila Nova de Gaia, Portugal; 2 Novartis Portugal, Porto Salvo, Portugal; 3 Department of Community Medicine, Information and Health Decision Sciences (MEDCIDS), Faculty of Medicine, University of Porto, Porto, Portugal; 4 MTG Research and Development Lab, Porto, Portugal; 5 Unidade Local de Saúde de Entre Douro e Vouga E.P.E., Santa Maria da Feira, Portugal; 6 Câmara Municipal de São João da Madeira, São João da Madeira, Portugal; 7 University of Coimbra Faculty of Medicine, Coimbra, Portugal; 8 University of Coimbra, Center for Innovative Biomedicine and Biotechnology (CIBB), Coimbra, Portugal; 9 University of Aveiro, Medical Sciences Department, Aveiro, Portugal

**Keywords:** cardiovascular diseases, community health planning, heart disease risk factors, occupational medicine, preventive medicine, primary prevention

## Abstract

**Introduction:**

In the face of escalating medical complexity, the development of simple strategies that allow the optimization of resources becomes more urgent than ever. This is particularly evident in cardiovascular diseases (CVD), where despite successful risk stratification and mitigation strategies, elevated mortality rates persist among socioeconomically disadvantaged groups. Efforts to prevent CVD events rely on early identification of at-risk individuals and timely implementation of risk-mitigating measures. Persistent challenges in health systems to identify and intervene with high-risk patients underline the growing importance of implementation science. Operating in the real-world context, this field allows to bridge the gap between identifying at-risk individuals and implementing tailored therapeutic strategies, facilitating the clinical assimilation of diagnostic and therapeutic advancements. RE-AIM (Reach, Effectiveness, Adoption, Implementation, Maintenance) framework was applied to structure and evaluate this implementation process.

**Methods and analysis:**

The CARDIOGAP + project emerges as a prospective solution to this challenge. By focusing on the industrial and manufacturing workforce, it seeks to achieve the early identification of high and very high-risk individuals through data routinely collected in Occupational Medicine (OM) appointments, which occur biennially (or annually for individuals aged 50 or over). Patients will be risk stratified according to the ESC guidelines using the data collected on the OM appointments, allowing a systematic and prompt recognition of individuals at elevated cardiovascular (CV) risk. The identified patients will be referred to a CV risk hospital consultation, where therapeutic interventions fostering adherence will be implemented. After the first hospital appointment, patients will be followed up to 1 year. As a pilot implementation study, sample size was determined by feasibility and precision considerations rather than by a formal power calculation for the exploratory 12-month risk-factor outcomes.

**Ethics and dissemination:**

This study was developed with reference to the SPIRIT (Standard Protocol Items: Recommendations for Interventional Trials) recommendations, adapted for a non-randomized implementation study, and in compliance with the Declaration of Helsinki and Good Pharmacoepidemiology Practice (GPP). Reporting of study results will follow the STROBE guidelines, ensuring ethical conduct and data integrity. Written informed consent will be obtained from all participants or their legal representatives. Confidentiality is safeguarded through de-identification and secure data storage. Results may be published or shared in public databases, following Novartis and ICMJE standards.

## 1 Introduction

Cardiovascular diseases (CVDs) remain the leading causes of death and disability worldwide and substantially contribute to loss of health and increasing health system costs. Several environmental, metabolic, and behavioral risk factors, including air pollution, high blood pressure, high levels of low-density lipoprotein cholesterol (LDL-C), kidney disfunction, tobacco smoking, among others, contribute to the development and establishment of CVD (1).

Atherosclerosis, a chronic inflammatory condition characterized by recruitment of white blood cells to the arterial wall, subsequent accumulation of lipids, and ultimate lesion formation, is the main pathophysiological process underlying clinical CVD (2). With time, the atherosclerotic plaque can become more fibrous and accumulate calcium mineral. Advanced atherosclerotic plaques can invade the arterial lumen, impeding blood flow and leading to myocardial infarctions (MI), stroke, and peripheral artery disease (3).

Atherosclerotic cardiovascular disease (ASCVD) incidence and mortality rates are declining in many countries in Europe, but it is still a major cause of morbidity and mortality (4). In fact, mortality data demonstrate that CVD remains the main cause of death in the European region. Furthermore, it is also important to acknowledge that there are important disparities across the different countries in the European region, as well as in the burden of CVD between men and women (5). Cardiovascular disease led to an annual toll of over 3.8 million deaths throughout Europe, comprising nearly 1.76 million deaths in men and exceeding 2 million deaths in women. This constituted 39% of all male deaths and 46% of all female deaths. In contrast, cancer, the second most prevalent cause of death in Europe, contributed to 24% of all male deaths and 20% of all female deaths (5). Although more women than men die from CVD in the region, higher age-standardized mortality and deaths in those aged <70 years demonstrate that CVD remains a greater burden among men in all countries. This observation substantiates the advocacy for alleviating the cardiovascular disease burden and mitigating disparities by instituting focused, evidence-driven interventions for both treatment and prevention across all Europe. In Portugal, atherosclerotic cardiovascular disease (ASCVD)-related costs amount to 1% of the Gross Domestic Product (6). In 2015, around 12% of the Portuguese population aged between 40 and 65 had a very high risk of developing a fatal cardiovascular event within the next 10 years. Thus, correctly identifying people with an elevated CV risk, at an early stage and the adoption of appropriate preventive strategies, is an important step to reduce CVD mortality in the Portuguese population (7).

While effective therapeutic strategies exist, much remains to be done in applying current knowledge more effectively and equitably in practice (3).

The development of simple strategies that allow the optimization of resources becomes more urgent than ever, particularly those focused on personalized problem solving through procedures that assure the clinical integration of diagnostic and therapeutic innovations. The use of preventive therapies in at­ risk individuals will likely demonstrate high cost­ effectiveness from a societal perspective (3). Implementation science, which tests, in the real world, the ability of health systems to deliver healthcare, is of increasing importance in this setting. To structure and evaluate this process, the present protocol adopts the RE-AIM framework (Reach, Effectiveness, Adoption, Implementation, Maintenance), a widely used implementation science framework for pragmatic, real-world health system interventions (8). RE-AIM dimensions are mapped onto the study's objectives in Section 2.1.3.

Considering CVD, it is essential to prevent the cardiovascular (CV) event from occurring, through the identification of individuals at risk and the early implementation of measures that mitigate this risk (9).

Despite the relatively easy identification of patients at risk and the effective therapies that exist, many of these patients end up entering the hospital not for the outpatient consultation, but through the emergency room with the CV event already established. This is, in many cases, due to the difficulty of the health system to identify and act on patients at greatest risk (10).

CARDIOGAP + proposes to provide an integrated response to this challenge, through:

- the early identification of patients at risk among those working at the industrial and manufacturing workforce, using data usually obtained in the Occupational Medicine (OM) consultation, as OM appointments are mandatory and take place every 2 years, or yearly for patients who are 50 or over, which allows for a regular follow-up of workers and an early and timely identification of patients at greater CV risk, even those that usually do not go to the Primary Care Physician and,
- the protocolization of referral to a hospital consultation, in a low-bureaucracy way upon the identification of very high CV risk patients by the OM physician who will refer the patient to the CV risk appointment where effective therapies that enhance therapeutic adherence will be thoroughly implemented.

In summary, CARDIOGAP + favors intervention strategies with several stakeholders aligned on a single mission: using the available resources and routinely collected data, and taking advantage of these existing, yet underused, mechanisms of referral to improve and promote the referral of very high CV risk patients to a CV risk appointment, thereby implementing simple and pragmatic strategies that cost-effectively avoid events, creating value for patients before they become ill.

## 2 Methods and analysis

### 2.1 Objectives

CARDIOGAP + aims to identify patients with elevated CV risk at OM consults and evaluate the effectiveness of the intervention (i.e., a simplified referral process of these patients by the OM of industrial/manufacturing companies for a cardiovascular risk hospital consultation) in CV health, through the assessment of the proportion of in-hospital consultation participation, adherence to therapy and the medium-term impact on risk factors.

#### 2.1.1 Primary objective

To implement a streamlined process to promote access to a CV risk hospital consultation, through the systematic identification of individuals with elevated CV risk in OM consultations carried out in industrial/manufacturing companies’ workers in the municipality of São João da Madeira.

#### 2.1.2 Secondary objectives

- Secondary objective 1: To assess the effective referral rate of identified patients with elevated CV risk in OM to the CV risk hospital consultation.
- Secondary objective 2: To assess the rate of hospital CV risk consults that effectively took place after the referral by the OM physician.
- Secondary objective 3: To measure the performance of the simplified referral process for a CV risk consultation, through the assessment of therapeutic adherence, and impact on the CV risk profile 12 months after the intervention.
- Secondary objective 4: To estimate the levels of Lipoprotein (a) in patients.
- Secondary objective 5: To estimate the prevalence of CV risk factors in workers in the industrial/manufacturing companies in the municipality of São João da Madeira, using the AHA Life's 8 system.
- Secondary objective 6: To assess the effective referral rate of patients to the primary care consultation.
- Secondary objective 7: To assess the effective application of the implementation strategies in the CARDIOGAP + project.

#### 2.1.3 Implementation science framework

This protocol is informed by the RE-AIM framework (Reach, Effectiveness, Adoption, Implementation, Maintenance), used to plan and evaluate pragmatic, real-world health system interventions. Primary and secondary objectives relate to RE-AIM dimensions as follows: Reach is addressed through the proportion of eligible OM attendees identified and screened (secondary objectives 1–2); Effectiveness is addressed through change in CV risk profile at 12 months (secondary objectives 3–4); Adoption is addressed through the referral rate by OM physicians and participating companies (secondary objectives 1 and 6); Implementation is addressed through fidelity to the referral protocol and consultation attendance (secondary objectives 2 and 7); and Maintenance, while outside the 18-month observation window of this pilot, will be discussed qualitatively as a direction for future, longer-term evaluation.

### 2.2 Study design and setting

**Figure 1:**
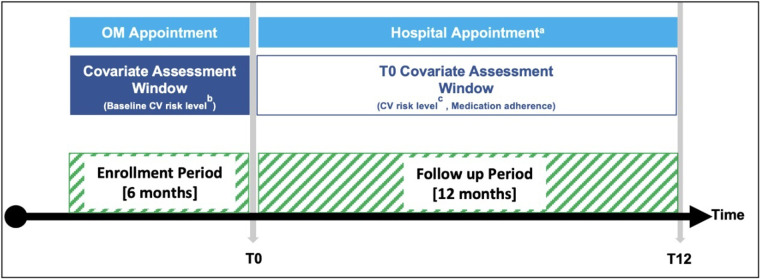
Overview of patient participation and data collection. OM: Occupational Medicine; CV: cardiovascular; T0: first hospital consultation; T12: 12-month hospital consultation; a) CV risk consultation and/or Smoking cessation consultation, when applicable; b) Risk prediction algorithms: SCORE2 for patients aged 40 years or more with HDL available; SCORE for patients aged 40 years or more without HDL available; c) Risk prediction algorithms: SCORE2 for patients aged 40 years or more. The enrollment phase includes the OM consultations occurring in the identified companies. The follow-up phase includes the hospital appointments occurring in ULSEDV.

Prospective pilot project for the integration of services, with a pragmatic design. Data collection lasts for 18 months in two distinct phases (Figure 1):

- A cross-sectional 6-month intervention phase to enroll patients and operationalize the primary objective and secondary objectives 1, 2 and 5. This is to implement a process to promote the access to cardiovascular risk hospital appointments, through the systematic identification of very high CV risk patients in appointments from Occupational Health carried out on workers from the industrial/manufacturing companies of the municipality of São João da Madeira.
- A longitudinal, clinical follow-up phase, lasting 12 months, to assess secondary objectives 3 through 6. This is to estimate pharmacological therapy adherence and the impact on cardiovascular risk profile after the intervention above mentioned.

#### 2.2.1 Eligibility criteria

The population of this study are the workers of the companies in the municipality of São João da Madeira included in the OM consultation of their company. To be eligible for this study, participants must meet the following criteria:

- Age ≥18 and ≤70 years
- Written informed consent obtained before participating in the study.
- Participants willing and able to complete the assessments as outlined in this study.
- Currently employed.

##### 2.2.1.1 Exclusion criteria

Only workers unwilling or unable to complete the assessments will be excluded.

Criteria of follow-up match the criteria for referral to a cardiovascular risk appointment by the Occupational Health physician. These criteria will be based on the Cardiovascular Prevention recommendations from 2021 of the European Society of Cardiology (ESC) and are as described in Table 1, regardless of whether there is a previous history of atherosclerotic disease. The combined use of the SCORE2 and SCORE algorithms reflects data availability in the OM setting: in the case of unavailability of HDL-cholesterol in the OM setting, the legacy SCORE algorithm is used as a pragmatic fallback, consistent with the flexibility allowed under the 2021 ESC guidelines (4, 11, 12).

**Table 1:** Criteria for referral to a cardiovascular risk appointment by the occupational health physician, based on the cardiovascular prevention recommendations from 2021 of the European Society of Cardiology (ESC).

*Markedly elevated single risk factor*
• Total cholesterol >310 mg/dL or LDL cholesterol >190 mg/dL
• Blood pressure >180/110 mmHg
• Moderate or severe chronic kidney disease, defined as eGFR <60 mL/min/1.73 m^2^ (CKD-EPI 2021 creatinine equation)

***Or very-high risk under SCORE2 stratification system***
• SCORE2 ≥ 7.5% if < 50y or SCORE2 ≥ 10% if 50−69y (18).

***Or high risk or very-high risk under SCORE stratification system***
• SCORE 5%–9.9%(high-risk); ≥10% (very high risk) (11).

#### 2.2.2 Sample size

Based on prior publications (7, 12, 13), it was estimated that up to 15% of the studied population has at least 1 of the inclusion criteria for referral (very high cardiovascular risk), so there is the expectation to perform 150 cardiovascular risk appointments in the first 6 months of the project (6–7 appointments/week, divided into two weekly periods of 4 h).

In this sense, instead of a sample size calculation, a precision estimation was calculated: for a confidence interval of 95%, a prevalence of 15% and 1000 persons included, a margin of error of 2.2% is estimated. This precision-based estimate addresses the primary objective and the descriptive prevalence outcomes (secondary objectives 1, 2 and 5), for which the expected sample provides adequate precision. As a pilot implementation study, CARDIOGAP + is not powered to test a pre-specified hypothesis regarding the magnitude of risk-factor change; these analyses are exploratory and hypothesis-generating, intended to inform the design and sample size of a future, adequately powered evaluation.

The sample size may be adjusted to the participation of physicians from Occupational Health and participating companies, with ULSEDV having the capacity to accommodate more referrals.

#### 2.2.3 Recruitment

The involvement of local stakeholders (Unidade Local de Saúde de Entre o Douro e Vouga – ULSEDV, São João da Madeira City Council and São João da Madeira Commercial and Industrial Association) in the protocolization of a specific referral network for patients with elevated cardiovascular risk in the influence area of the ULSEDV, based on the universality and obligatory nature of Occupational Medicine consultations, was the adopted strategy for the identification of the participating companies and population’s recruitment.

#### 2.2.4 Data collection

Participants will be assessed in three time points:

- at baseline, at their working company by Occupational Safety and Health Technician and by OM doctor
- at T0, at hospital centre by a medical doctor, for participants referred by the OM doctor based on their CV-risk level
- at T12, at hospital centre by a medical doctor, 12 months after T0.

Study variables to be used in this study, and related definitions, are described in the following subsections. Table 2 shows the set of data that will be collected and variable features. The main study variables concern demographics, habits, laboratory testing, comorbidities, medications and destination are listed below in more detail.

**Table 2:** Data collection – variables and corresponding features.

Variable	Label	Evaluation period
Inclusion Criteria Check		
consentimento_informado	Signed informed consent	Baseline
avaliacao_possivel	Participant willing and able to complete the assessments	Baseline
Demographics and disease history		
ano_de_nascimento	Birth year	Baseline
sexo	Sex	Baseline
tabagismo_status	Smoking status	Baseline, T0, T12
dieta	Enter Diet Data?^a^	Baseline
sono	On average, how many hours of sleep do you get in a 24-hour period?^b^	Baseline
exercicio_fisico	How many minutes of moderate (or greater) intensity activity per week?^b^	Baseline
drc_moderada_grave	Presence of Moderate or Severe Chronic Kidney Disease	Baseline, T0, T12
dm	Presence of Diabetes Mellitus	Baseline, T0, T12
historia_evento_cv	History of Cardiovascular Event	Baseline, T0, T12
especificacao_eventos_cv	Specification of Cardiovascular Events	Baseline, T0, T12
outros_antecedentes	Other Pathological History	Baseline, T0, T12
Diet Questionnaire		
dieta_porcoes_vegetais_semana	How many servings of vegetables do you consume per week?	Baseline
dieta_porcoes_carne_vermelha_semana	Servings of Red Meat per Week	Baseline
dieta_porcoes_manteiga_creme_semana	How many servings of butter or cream do you consume per week?	Baseline
dieta_porcoes_graos_integrais_dia	How many servings of whole grains do you consume per day?	Baseline
dieta_consumo_fastfood_semana	Times Consuming Fast Food Meals per Week	Baseline
por_es_de_frutas_por_semana	How many servings of fruit do you consume per week?	Baseline
dieta_peixe_semana	How many servings of fish or shellfish/seafood do you consume per week?	Baseline
dieta_feijoes_semana	How many servings of beans do you consume per week?	Baseline
dieta_doces_semana	How many servings of commercial sweets, candy bars, pastries, cookies, or cakes do you consume per week?	Baseline
dieta_bebidas_acucaradas_semana	How many servings of sugar-sweetened beverages do you drink per week?	Baseline
Usual Medication		
anti_dislipidemico	Anti-dyslipidemic	Baseline, T0, T12
anti_hta	Anti-hypertensive	Baseline, T0, T12
anti_agregantes	Anti-platelet	Baseline, T0, T12
farmaco_sem_medicacao	Without Usual Medication	T0, T12
farmaco_mh_dci	Drug (Previous Usual Medication) - International Nonproprietary Name (INN)	T0, T12
farmaco_mh_dose	Drug (Previous Usual Medication) - Dose	T0, T12
outro_farmaco	Insert another drug	T0, T12
Prescribed Medication		
intervencao	Was a new drug prescribed as a result of this assessment?	T0, T12
farmaco_intervencao_dci	Newly Prescribed Drug - International Nonproprietary Name (INN)	T0, T12
farmaco_intervencao_dose	Newly Prescribed Drug - Dose	T0, T12
outro_farmaco_intervencao	Insert another drug?	T0,T12
Clinical Evaluation		
ta_sist	Systolic Blood Pressure (mmHg)	Baseline, T0, T12
ta_diast	Diastolic Blood Pressure (mmHg)	Baseline, T0, T12
fc	Heart Rate (bpm)	Baseline, T0, T12
peso	Weight (kg)	Baseline, T0, T12
altura	Height (cm)	Baseline, T0, T12
Laboratory testing		
data_avaliacao_analitica	Date of Analytical Assessment	Baseline, T0, T12
colesterol_total	Total Cholesterol (mg/dL)	Baseline, T0, T12
ldl_c	LDL-C (mg/dL)	Baseline, T0, T12
hdl_c	HDL-C (mg/dL)	Baseline, T0, T12
triglicerideos	Triglycerides (mg/dL)	Baseline, T0, T12
hba1c	HbA1c (%)	Baseline, T0, T12
creatinina	Creatinine (mg/dL)	Baseline, T0, T12
egfr	Estimated Glomerular Filtration Rate (mL/min/1.73m^2^)	Baseline, T0, T12
ureia	Urea (mg/dL)	Baseline
lp_a	Lipoprotein (a)	T0
apo_a	Apolipoprotein A	T0, T12
apo_b	Apolipoprotein B	T0, T12
Destination		
destino_consulta_mo	Destination of the Participant	Baseline
destino_final_hospitalar	Destination After 12 Months of Hospital Follow-Up	T12
Medication adherence questionnaire		
qat_1_toma_hora	Do you always take your medication at the appropriate time?	T12
qat_2_sentir_mal	When you feel bad, have you ever discontinued taking your medication?	T12
qat_3_esquecer	Have you ever forgotten to take your medication?	T12
qat_4_esquecer_fds	Have you ever forgotten to take your medication during the weekend?	T12
qat_5_falha_semana	In the last week, how many times did you failed to take your prescribed dose?	T12
qat_6_dias_sem_medicacao	Since your last visit, how many whole days have gone in which you did not take your medication?	T12

a Indicates whether information about the participant's diet will be entered. This variable is mandatory for participants under 40 years old.

b Question from Life's Essential 8 questionnaire (14).

#### 2.2.5 Inclusion criteria check

Inclusion criteria will be checked by Occupational Safety and Health Technician prior to OM appointment.

#### 2.2.6 Demographics and disease history

At baseline, demographics and disease history will be recorded on paper by the Occupational Safety and Health Technician during OM appointments. The study coordinator will later collect these records, assign a participant number to replace the SNS number in the eCRF, and generate a key pair to link the SNS number with the participant number. At T0 and T12, smoking habits and disease history will also be initially recorded on paper by the hospital medical doctor, and the same process of replacing the SNS number with a participant number and recording the key pair will be followed for entry into the eCRF.

#### 2.2.7 Diet questionnaire

Diet questions from Life's Essential 8 questionnaire (14) will be mandatory for participants under 40 years old. These data will be recorded by Occupational Safety and Health Technician before the OM appointment.

#### 2.2.8 Usual medication

At baseline, usual medication will be recorded on paper by the Occupational Safety and Health Technician during OM appointments, with the data later entered into the eCRF. At T0 and T12, usual medication will be recorded on paper by the hospital medical doctor, following the same process for entry into the eCRF.

#### 2.2.9 Prescribed medication

At T0 and T12, any newly prescribed medication will be recorded on paper by the hospital medical doctor after the clinical evaluation, with the data later entered into the eCRF.

#### 2.2.10 Clinical evaluation

At baseline, clinical evaluation will be recorded on paper by the Occupational Safety and Health Technician during OM appointments, with the data later entered into the eCRF. At T0 and T12, clinical evaluation will be recorded by the hospital medical doctor and entered into the eCRF.

#### 2.2.11 Laboratory testing

At baseline, laboratory testing results will be recorded on paper by the Occupational Safety and Health Technician before OM appointments, with the data later entered into the eCRF by the study coordinator. At T0 and T12, laboratory results will be recorded by the hospital medical doctor and later entered into the eCRF by the study coordinator.

#### 2.2.12 Destination

These variables are related to the referral given to the participant after the clinical evaluation. At the baseline, the OM doctor indicates whether advice was given, referred to Family Medicine, or to a hospital; at T12, the hospital medical doctor indicates whether participant was discharged or continue with hospital follow-up.

#### 2.2.13 Medication adherence questionnaire

At T12, medication adherence questions from the adapted Simplified Medication Adherence Questionnaire (SMAQ) will be answered on paper and later entered into the eCRF by the study coordinator. The adapted SMAQ was selected because it is a brief, easily administered instrument suitable for routine clinical practice and implementation studies involving patients receiving multiple long-term medications. Although originally validated in renal transplant recipients, it has been used as a pragmatic measure of medication adherence across chronic disease settings (15, 16).

### 2.3 Data management and analysis

An eCRF will be developed by the CRO for data collection, aligned with study's specific objectives and researchers’ preferences, and will be hosted by ULSEDV on the institution's approved RedCAP system. During OM and hospital appointments, data will be initially recorded on paper, including the SNS number and visit date, and stored alongside other clinical records under the same security measures. The study coordinator will periodically collect these paper records from the study sites, assign a participant number to replace the SNS number in the electronic records and generate a key pair to link the SNS number with the participant number. The coordinator will then enter the collected data into the eCRF and ensure the accuracy of data. The key pair and original paper source documents will be stored at ULSEDV in compliance with institutional regulations. The date of each visit will also be recorded.

The sponsor, NVS, and the designated CRO, MTG Research & Development Lab, will have oversight of the data management process but will not directly handle any patient-identifiable information. Primary statistical analyses will be conducted by the CRO and ULSEDV-affiliated investigators independently of Novartis Portugal. Novartis Portugal will have no access to patient-level data, will not perform or direct the statistical analyses, and will not have authority over the interpretation of results or the decision to publish, consistent with ICMJE and GPP recommendations on sponsor independence.

#### 2.3.1 Statistical methods

To address the multifaceted endpoints of this study (Table 3), a combination of statistical techniques will be employed. Descriptive statistics will be calculated to assess various CV risk factors. Continuous variables will be summarized using n, mean, median, standard deviation, minimum, maximum and 25th and 75th percentiles. Categorical variables will be summarized using frequency counts and percentages. Missing data will be reported using frequency counts and percentages and assessed for pattern and extent; where data are judged to be missing at random, multiple imputation will be used as the primary approach. Outliers will be identified using standard distributional criteria, and treated in consideration of their impact on the analysis.

**Table 3:** Cardiogap + endpoints.

Endpoint Description
1. Proportion (%) of patients who meet the follow-up criteria for CV risk hospital consultation identified in OM consultation: Descriptive statistics will be used to calculate the percentage of patients who meet the follow-up criteria for CV risk hospital consultation over all patients attending OM consultations.
2. Proportion (%) of High CV risk individuals identified in OM consultation: Descriptive statistics will be used to calculate the percentage of High CV patients over all patients attending OM consultations.
3. Proportion (%) of Very High CV risk individuals identified in OM consultation: Descriptive statistics will be used to calculate the percentage of Very High CV patients over all patients attending OM consultations.
4. Proportion (%) of patients referred to Hospital CV Risk Consultation, overall and stratified by risk category: Descriptive statistics will be used to calculate the percentage of patients referred to Hospital CV Risk Consultation, reported (a) over allpatients meeting follow-up criteria, (b) over High CV risk patients only, and (c) over Very High CV risk patients only, identified in OM consultation.
5. Proportion (%) of Hospital CV Risk Consultation Participation after Referral by OM: Descriptive statistics will be used to calculate the percentage of individuals referred from OM consultations who participate in hospital CV risk consultations over all patients referred to hospital consultations.
6. Level of Adherence to Medication Prescribed in the Hospital CV Risk Consultation: Descriptive statistics will assess the adherence levels, measured by SMAQ among patients prescribed medications in the hospital CV risk consultations.
7. Proportion of Patients Who Attended Smoking Cessation Consultation: Descriptive statistics will determine the proportion of patients attending smoking cessation consultations over patients who were referred and participated in hospital CV risk consultation.
8. Change in distribution from Baseline in Various CV Risk Factors after 12 Months: Appropriate statistical methods will be used to calculate the distribution change from baseline (first hospital consultation) for the following CV risk factors after the use of this simplified referral process.
a. LDL-C
b. Blood pressure (SBP and DBP)
c. Moderate or severe chronic kidney disease
d. Smoking status
e. HbA1c
f. BMI
Statistical methods for this endpoint are specified in Section 2.3.1.
9. Distribution of Lipoprotein (a) at the first hospital consult: Descriptive statistics will assess the distribution of Lipoprotein (a) at the first hospital consult.
10. Prevalence of CV Risk Factors in the industrial/manufacturing in São João da Madeira: Appropriate statistical methods will be used to estimate the prevalence of CV risk factors within the industrial/manufacturing companies, including the levels of Lipoprotein (a) among very high CV risk individuals.
11. Proportion (%) of Identified Patients Referenced to GP Consultation: Descriptive statistics will be used to calculate the percentage of patients referred to GP consultations.

For the change in CV risk factors from baseline/T0 to T12, repeated continuous measures will be analyzed using linear mixed-effects models (or generalized estimating equations, as appropriate to the outcome distribution) with a random intercept per participant to account for within-subject correlation across the baseline, T0 and T12 assessments. Binary or categorical repeated measures will be analyzed using generalized estimating equations with a logit link. Models will be adjusted *a priori* for age, sex, baseline CV risk category, and baseline value of the outcome variable; additional covariates identified as clinically relevant confounders may be added in sensitivity analyses.

#### 2.3.2 Data monitoring

Data entry is carried out by the treating physician and study nurses/coordinators on the paper version of the CRF. The final version of the eCRF web application is located on a secure server managed by the ULSEDV. Data entry staff will not gain access to the eCRF until they have been trained. During data entry programmed validations will be in place to check the data for plausibility and accuracy.

The development of analytical code follows a structured process involving both self-review and independent review. Code is managed using version control through Git with a remote repository on an access-controlled GitHub repository. This ensures that all changes are tracked, and the code is systematically reviewed before deployment, ensuring the integrity of the analysis.

ULSEDV will ensure that data quality processes are followed, including verification of completeness and accuracy of eCRFs data, based on the paper records collected at the study sites. Quality checks will be conducted by the CRO using automated analytical packages, ensuring data integrity while complying with access restrictions.

The study coordinator is responsible for retrieving, maintaining, and inputting data from the source documents for each patient in the study which contain demographic and medical information, as well as the results of any other tests or assessments, and ensuring that all information entered into the eCRF is traceable to these source documents. Any discrepancies between the source documents and the eCRF must be documented and explained. The study-related records will be retained for 3 years after the study's conclusion.

Any deviation from the protocol will be documented in an addendum and duly submitted to the local Ethics Committee.

### 2.4 Strengths and limitations

CARDIOGAP + relies on a low-bureaucracy referral pathway designed for real-world adoption using mandatory occupational health infrastructure to screen a working-age population often missed by primary-care-based cardiovascular screening. In this context, three main limitations should be noted: Possible underestimation of true population-level risk (a “healthy worker effect”) due to restricting enrollment to currently employed individuals (since unemployed, informally employed, retired, and long-term sick-leave workers may carry a higher CV risk burden); Absence of power for a confirmatory test of 12-month risk-factor change (exploratory outcome); Barriers to hospital consultation adherence, which may be limited by shift workers’ difficulty attending hospital appointments, transportation constraints, low risk-perception among asymptomatic individuals, and gaps in OM-hospital communication

However, it should be noted that the low-bureaucracy design partially mitigates all these constraints and limitations, and their impact will be assessed empirically (secondary objectives 1, 2, 3, 7).

## 3 Ethics and dissemination

### 3.1 Research ethics approval

This study was reviewed and approved by the Ethics Committee of ULSEDV. Compliance with Novartis and regulatory standards provides assurance that the rights, safety, and well-being of patients participating in non-interventional studies are protected (consistent with the principles that have their origin in the Declaration of Helsinki) and that the study data are credible and responsibly reported.

This study was designed and shall be implemented with reference to the SPIRIT (Standard Protocol Items: Recommendations for Interventional Trials) guidance for protocol reporting, adapted for a non-randomized implementation pilot, and reported in accordance with the Guidelines for Good Pharmacoepidemiology Practices (GPP) of the International Society for Pharmacoepidemiology (17), the STROBE (Strengthening the Reporting of Observational Studies in Epidemiology) guidelines (18), and with the ethical principles laid down in the Declaration of Helsinki.

### 3.2 Consent

The Occupational Safety and Health Technician will provide the informed consent form to the participant on-site and store it alongside the other clinical records. The study coordinator will later collect the consent form, along with the other paper versions of the CRF, and keep it in the ULSEDV. A signed copy will be given to the participant.

Eligible patients may only be included in the study after providing written (witnessed, where required by law or regulation), IRB/EC-approved informed consent, or, if incapable of doing so, after such consent has been provided by a legally acceptable representative of the patient. In cases where the patient's representative gives consent, the patient should be informed about the study to the extent possible given his/her understanding. If the patient is capable of doing so, he/she should assent by personally signing and dating the written informed consent document or a separate assent form. Informed consent must be obtained before any data are collected. The process of obtaining informed consent should be documented in the patient source documents.

Treating physicians or other involved medical professionals will be provided in a separate document a proposed informed consent form that complies with the Declaration of Helsinki principle and regulatory requirements and is considered appropriate for this study.

It's important to note that participation in this study will not result in any alterations to the standard treatment provided to the patients. Therefore, no risks or discomforts are expected for participants due to their involvement in this research. Participation is voluntary and completely free for all individuals involved. Participants have the freedom to withdraw from the project at any time, without any adverse consequences or impact on their care.

### 3.3 Confidentiality

The SNS number recorded on paper will be replaced by a unique participant number in the eCRF. A key pair will be stored to link the SNS number with the participant number. The original paper records will be stored at the study sites under the same conditions as other clinical records. The eCRF will be hosted within the ULSEDV infrastructure and managed by ULSEDV, ensuring all data processing, storage, and access comply with the institutional regulations. Access to identifiable information will be restricted to healthcare professionals directly involved in patient care and staff designated by ULSEDV. For all other parties involved in the research, access is restricted to aggregated results generated by automated analytical packages within ULSEDV's infrastructure and provided by ULSEDV.

### 3.4 Dissemination policy

The results of this non-interventional study may be either submitted for publication and/or posted in a publicly accessible database of results. Publications will comply with internal Novartis standards and the International Committee of Medical Journal Editors (ICMJE) guidelines.
